# Supplementary material for: OxInflammation Affects Transdifferentiation to Myofibroblasts, Prolonging Wound Healing in Diabetes: A Systematic Review
Source: Int J Mol Sci. 2024 Aug 19;25(16):8992. doi: 10.3390/ijms25168992 (PMC11354661; doi:10.3390/ijms25168992)
Supplement: Supplementary file 1 [file ijms-25-08992-s001.zip › ijms-3121675-supplementary.pdf]

Supplementary material

Table S1

Supporting Information

S1 Table Complete search strategy filters and number of studies recovery and data bases is PubMed and SCOPUS and Web of Science.

Data base	Descriptors	Items Found	Time	Date
P U B M E D	#1 Wound Healing" [MeSH terms] OR " Wound Healing "[TIAB]	169 961	13:41:45	04/10/2021
	#2 "myofibroblasts"[MeSH Terms] OR "myofibroblasts"[TIAB]	11 312	13:42:13	04/10/2021
	# 3 ("Skin" [MeSH terms] OR "Dermis" [MeSH terms] OR "Granulation Tissue"[MeSH terms] OR "Epidermis"[MeSH terms] OR "Keratinocytes"[MeSH terms] OR "Integumentary System"[MeSH terms] OR "Dermatology"[MeSH terms] OR "Dermoscopy"[MeSH terms] OR "Wounds and Injuries"[MeSH terms] OR "Fibrosis"[MeSH terms] OR "Skin injuries"[TIAB] OR "Skin fibrosis"[TIAB] OR "Skin scars"[TIAB] OR "Cicatrix"[MeSH terms])	1 364 342	13:42:35	04/10/2021
	#4 Animal ("animal experimentation"[MeSH Terms] OR "models, animal"[MeSH Terms] OR "invertebrates"[MeSH Terms] OR "Animals"[Mesh:noexp] OR "animal population groups"[MeSH Terms] OR "chordata"[MeSH Terms:noexp] OR "chordata, nonvertebrate"[MeSH Terms] OR "vertebrates"[MeSH Terms:noexp] OR "amphibians"[MeSH Terms] OR "birds"[MeSH Terms] OR "fishes"[MeSH Terms] OR "reptiles"[MeSH Terms] OR "mammals"[MeSH	7 273 832	13:43:13	04/10/2021

	Terms:noexp] OR "primates"[MeSH Terms:noexp] OR "artiodactyla"[MeSH Terms] OR "carnivora"[MeSH Terms] OR "cetacea"[MeSH Terms] OR "chiroptera"[MeSH Terms] OR "elephants"[MeSH Terms] OR "hyraxes"[MeSH Terms] OR "insectivora"[MeSH Terms] OR "lagomorpha"[MeSH Terms] OR "marsupialia"[MeSH Terms] OR "monotremata"[MeSH Terms] OR "perissodactyla"[MeSH Terms] OR "rodentia"[MeSH Terms] OR "scandentia"[MeSH Terms] OR "sirenia"[MeSH Terms] OR "xenarthra"[MeSH Terms] OR "haplorhini"[MeSH Terms:noexp] OR "strepsirhini"[MeSH Terms] OR "platyrrhini"[MeSH Terms] OR "tarsii"[MeSH Terms] OR "catarrhini"[MeSH Terms:noexp] OR "cercopithecidae"[MeSH Terms] OR "hylobatidae"[MeSH Terms] OR "hominidae"[MeSH Terms:noexp] OR "gorilla gorilla"[MeSH Terms] OR "pan paniscus"[MeSH Terms] OR "pan troglodytes"[MeSH Terms] OR "pongo pygmaeus"[MeSH Terms]) OR<br><br>((animals[TIAB] OR animal[TIAB] OR mice[TIAB] OR mus[TIAB] OR mouse[TIAB] OR murine[TIAB] OR woodmouse[TIAB] OR rats[TIAB] OR rat[TIAB] OR murinae[TIAB] OR muridae[TIAB] OR cottonrat[TIAB] OR cottonrats[TIAB] OR hamster[TIAB] OR hamsters[TIAB] OR cricetinae[TIAB] OR rodentia[TIAB] OR rodent[TIAB] OR rodents[TIAB] OR pigs[TIAB] OR pig[TIAB] OR swine[TIAB] OR swines[TIAB] OR piglets[TIAB] OR piglet[TIAB] OR boar[TIAB] OR boars[TIAB] OR "sus scrofa"[TIAB] OR ferrets[TIAB] OR ferret[TIAB] OR polecat[TIAB] OR polecats[TIAB] OR "mustela putorius"[TIAB] OR "guinea pigs"[TIAB] OR "guinea pig"[TIAB] OR cavia[TIAB] OR callithrix[TIAB] OR marmoset[TIAB] OR marmosets[TIAB] OR cebuella[TIAB] OR hapale[TIAB] OR octodon[TIAB] OR chinchilla[TIAB] OR chinchillas[TIAB] OR			

	gerbillinae[TIAB] OR gerbil[TIAB] OR gerbils[TIAB] OR jird[TIAB] OR jirds[TIAB] OR merione[TIAB] OR meriones[TIAB] OR rabbits[TIAB] OR rabbit[TIAB] OR hares[TIAB] OR hare[TIAB] OR diptera[TIAB] OR flies[TIAB] OR fly[TIAB] OR dipteral[TIAB] OR drosphila[TIAB] OR drosophilidae[TIAB] OR cats[TIAB] OR cat[TIAB] OR carus[TIAB] OR felis[TIAB] OR nematoda[TIAB] OR nematode[TIAB] OR nematoda[TIAB] OR nematode[TIAB] OR nematodes[TIAB] OR sipunculida[TIAB] OR dogs[TIAB] OR dog[TIAB] OR canine[TIAB] OR canines[TIAB] OR canis[TIAB] OR sheep[TIAB] OR sheeps[TIAB] OR mouflon[TIAB] OR mouflons[TIAB] OR ovis[TIAB] OR goats[TIAB] OR goat[TIAB] OR capra[TIAB] OR capras[TIAB] OR rupicapra[TIAB] OR chamois[TIAB] OR haplorhini[TIAB] OR monkey[TIAB] OR monkeys[TIAB] OR anthropoidea[TIAB] OR anthropoids[TIAB] OR saguinus[TIAB] OR tamarin[TIAB] OR tamarins[TIAB] OR leontopithecus[TIAB] OR hominidae[TIAB] OR ape[TIAB] OR apes[TIAB] OR pan[TIAB] OR paniscus[TIAB] OR "pan paniscus"[TIAB] OR bonobo[TIAB] OR bonobos[TIAB] OR troglodytes[TIAB] OR "pan troglodytes"[TIAB] OR gibbon[TIAB] OR gibbons[TIAB] OR siamang[TIAB] OR siamangs[TIAB] OR nomascus[TIAB] OR symphalangus[TIAB] OR chimpanzee[TIAB] OR chimpanzees[TIAB] OR prosimians[TIAB] OR "bush baby"[TIAB] OR prosimian[TIAB] OR bush babies[TIAB] OR galagos[TIAB] OR galago[TIAB] OR pongidae[TIAB] OR gorilla[TIAB] OR gorillas[TIAB] OR pongo[TIAB] OR pygmaeus[TIAB] OR "pongo pygmaeus"[TIAB] OR orangutans[TIAB] OR pygmaeus[TIAB] OR lemur[TIAB] OR lemurs[TIAB] OR lemuridae[TIAB] OR horse[TIAB] OR horses[TIAB] OR pongo[TIAB] OR equus[TIAB] OR cow[TIAB] OR calf[TIAB] OR bull[TIAB] OR chicken[TIAB] OR chickens[TIAB] OR gallus[TIAB] OR quail[TIAB] OR bird[TIAB]			

	OR birds[TIAB] OR quails[TIAB] OR poultry[TIAB] OR poultries[TIAB] OR fowl[TIAB] OR fowls[TIAB] OR reptile[TIAB] OR reptilia[TIAB] OR reptiles[TIAB] OR snakes[TIAB] OR snake[TIAB] OR lizard[TIAB] OR lizards[TIAB] OR alligator[TIAB] OR alligators[TIAB] OR crocodile[TIAB] OR crocodiles[TIAB] OR turtle[TIAB] OR turtles[TIAB] OR amphibian[TIAB] OR amphibians[TIAB] OR amphibia[TIAB] OR frog[TIAB] OR frogs[TIAB] OR bombina[TIAB] OR salientia[TIAB] OR toad[TIAB] OR toads[TIAB] OR "epidalea calamita"[TIAB] OR salamander[TIAB] OR salamanders[TIAB] OR eel[TIAB] OR eels[TIAB] OR fish[TIAB] OR fishes[TIAB] OR pisces[TIAB] OR catfish[TIAB] OR catfishes[TIAB] OR siluriformes[TIAB] OR arius[TIAB] OR heteropneustes[TIAB] OR sheatfish[TIAB] OR perch[TIAB] OR perches[TIAB] OR percidae[TIAB] OR perca[TIAB] OR trout[TIAB] OR trouts[TIAB] OR char[TIAB] OR chars[TIAB] OR salvelinus[TIAB] OR "fathead minnow"[TIAB] OR minnow[TIAB] OR cyprinidae[TIAB] OR carps[TIAB] OR carp[TIAB] OR zebrafish[TIAB] OR zebrafishes[TIAB] OR goldfish[TIAB] OR goldfishes[TIAB] OR guppy[TIAB] OR guppies[TIAB] OR chub[TIAB] OR chubs[TIAB] OR tinca[TIAB] OR barbels[TIAB] OR barbus[TIAB] OR pimephales[TIAB] OR promelas[TIAB] OR "poecilia reticulata"[TIAB] OR mullet[TIAB] OR mullets[TIAB] OR seahorse[TIAB] OR seahorses[TIAB] OR mugil curema[TIAB] OR atlantic cod[TIAB] OR shark[TIAB] OR sharks[TIAB] OR catshark[TIAB] OR anguilla[TIAB] OR salmonid[TIAB] OR salmonids[TIAB] OR whitefish[TIAB] OR whitefishes[TIAB] OR salmon[TIAB] OR salmons[TIAB] OR sole[TIAB] OR solea[TIAB] OR "sea lamprey"[TIAB] OR lamprey[TIAB] OR lampreys[TIAB] OR pumpkinseed[TIAB] OR sunfish[TIAB] OR sunfishes[TIAB] OR tilapia[TIAB] OR			

	tilapias[TIAB] OR turbot[TIAB] OR turbots[TIAB] OR flatfish[TIAB] OR flatfishes[TIAB] OR sciuridae[TIAB] OR squirrel[TIAB] OR squirrels[TIAB] OR chipmunk[TIAB] OR chipmunks[TIAB] OR suslik[TIAB] OR susliks[TIAB] OR vole[TIAB] OR voles[TIAB] OR lemming[TIAB] OR lemmings[TIAB] OR muskrat[TIAB] OR muskrats[TIAB] OR lemmus[TIAB] OR otter[TIAB] OR otters[TIAB] OR marten[TIAB] OR martens[TIAB] OR martes[TIAB] OR weasel[TIAB] OR badger[TIAB] OR badgers[TIAB] OR ermine[TIAB] OR mink[TIAB] OR minks[TIAB] OR sable[TIAB] OR sables[TIAB] OR gulo[TIAB] OR gulos[TIAB] OR wolverine[TIAB] OR wolverines[TIAB] OR minks[TIAB] OR mustela[TIAB] OR llama[TIAB] OR llamas[TIAB] OR alpaca[TIAB] OR alpacas[TIAB] OR camelid[TIAB] OR camelids[TIAB] OR guanaco[TIAB] OR guanacos[TIAB] OR chiroptera[TIAB] OR chiropteras[TIAB] OR bat[TIAB] OR bats[TIAB] OR fox[TIAB] OR foxes[TIAB] OR iguana[TIAB] OR iguanas[TIAB] OR xenopus laevis[TIAB] OR parakeet[TIAB] OR parakeets[TIAB] OR parrot[TIAB] OR parrots[TIAB] OR donkey[TIAB] OR donkeys[TIAB] OR mule[TIAB] OR mules[TIAB] OR zebra[TIAB] OR zebras[TIAB] OR shrew[TIAB] OR shrews[TIAB] OR bison[TIAB] OR bisons[TIAB] OR buffalo[TIAB] OR buffaloes[TIAB] OR deer[TIAB] OR deers[TIAB] OR bear[TIAB] OR bears[TIAB] OR panda[TIAB] OR pandas[TIAB] OR "wild hog"[TIAB] OR "wild boar"[TIAB] OR fitchew[TIAB] OR fitch[TIAB] OR beaver[TIAB] OR beavers[TIAB] OR jerboa[TIAB] OR jerboas[TIAB] OR capybara[TIAB] OR capybaras[TIAB]) NOT medline[subset])			
	Total: #1 and #2 and #3 and #4	706	13:43:51	04/10/2021

Data base	Descriptors	Items Found	Time	Date
S C O P U S	#1 TITLE-ABS-KEY (“Wound Healing”)	182 349	14:52:15	04/10/2021
	#2 TITLE-ABS-KEY(“myofibroblasts”)	11 935	14:53:19	04/10/2021
	#3 (TITLE-ABS-KEY(Skin) OR TITLE-ABS-KEY(Dermis) OR TITLE-ABS-KEY (“Granulation Tissue”) OR TITLE-ABS-KEY(Epidermis) OR TITLE-ABS-KEY(Keratinocyte*) OR TITLE-ABS-KEY (Integumentary System) OR TITLE-ABS-KEY(Dermatology) OR TITLE-ABS-KEY(Dermoscopy) OR TITLE-ABS-KEY(Skin wounds) OR TITLE-ABS-KEY(Skin injuries) OR TITLE-ABS-KEY(Skin fibrosis) OR TITLE-ABS-KEY(Skin scar*) OR (Skin cicatrix))	1 393 394	14:54:16	04/10/2021
	Total: #1 and #2 and #3	814	14:54:45	04/10/2021
	**Keywords Animal Model**	260	14:55:20	04/10/2021
**Data base**	**Descriptors**	**Items Found**	**Time**	**Date**
W E B of S C I E N C E	#1 TS=(Wound Healing)	**89 211**	**15:01:15**	**04/10/2021**
	#2 TS= myofibroblasts	**11 839**	**15:04:10**	**04/10/2021**
	#3 TS=Skin OR TS=Dermis OR TS=Granulation tissue OR TS=Epidermis OR TS=Keratinocyte OR TS=Integumentary system OR TS=Dermatology OR TS=Dermoscopy OR TS=Skin wounds OR TS=Skin injuries OR TS=Skin fibrosis OR TS=Skin scar OR TS=Skin cicatrix	**718 309**	**15:04:28**	**04/10/2021**
	#4 Animal	**6 491 034**	**15:08:36**	**04/10/2021**

	("animal experimentation"[MeSH Terms] OR "models, animal"[MeSH Terms] OR "invertebrates"[MeSH Terms] OR "Animals"[Mesh:noexp] OR "animal population groups"[MeSH Terms] OR "chordata"[MeSH Terms:noexp] OR "chordata, nonvertebrate"[MeSH Terms] OR "vertebrates"[MeSH Terms:noexp] OR "amphibians"[MeSH Terms] OR "birds"[MeSH Terms] OR "fishes"[MeSH Terms] OR "reptiles"[MeSH Terms] OR "mammals"[MeSH Terms:noexp] OR "primates"[MeSH Terms:noexp] OR "artiodactyla"[MeSH Terms] OR "carnivora"[MeSH Terms] OR "cetacea"[MeSH Terms] OR "chiroptera"[MeSH Terms] OR "elephants"[MeSH Terms] OR "hyraxes"[MeSH Terms] OR "insectivora"[MeSH Terms] OR "lagomorpha"[MeSH Terms] OR "marsupialia"[MeSH Terms] OR "monotremata"[MeSH Terms] OR "perissodactyla"[MeSH Terms] OR "rodentia"[MeSH Terms] OR "scandentia"[MeSH Terms] OR "sirenia"[MeSH Terms] OR "xenarthra"[MeSH Terms] OR "haplorhini"[MeSH Terms:noexp] OR "strepsirhini"[MeSH Terms] OR "platyrrhini"[MeSH Terms] OR "tarsii"[MeSH Terms] OR "catarrhini"[MeSH Terms:noexp] OR "cercopithecidae"[MeSH Terms] OR "hylobatidae"[MeSH Terms] OR "hominidae"[MeSH Terms:noexp] OR "gorilla gorilla"[MeSH Terms] OR "pan paniscus"[MeSH Terms] OR "pan troglodytes"[MeSH Terms] OR "pongo pygmaeus"[MeSH Terms]) or ((animals[TIAB] OR animal[TIAB] OR mice[TIAB] OR mus[TIAB] OR mouse[TIAB] OR murine[TIAB] OR woodmouse[TIAB] OR rats[TIAB] OR rat[TIAB] OR murinae[TIAB] OR muridae[TIAB] OR cottonrat[TIAB] OR cottonrats[TIAB] OR hamster[TIAB] OR hamsters[TIAB] OR cricetinae[TIAB] OR rodentia[TIAB] OR rodent[TIAB] OR rodents[TIAB] OR pigs[TIAB] OR pig[TIAB]			

	OR swine[TIAB] OR swines[TIAB] OR piglets[TIAB] OR piglet[TIAB] OR boar[TIAB] OR boars[TIAB] OR "sus scrofa"[TIAB] OR ferrets[TIAB] OR ferret[TIAB] OR polecat[TIAB] OR polecats[TIAB] OR "mustela putorius"[TIAB] OR "guinea pigs"[TIAB] OR "guinea pig"[TIAB] OR cavia[TIAB] OR callithrix[TIAB] OR marmoset[TIAB] OR marmosets[TIAB] OR cebuella[TIAB] OR hapale[TIAB] OR octodon[TIAB] OR chinchilla[TIAB] OR chinchillas[TIAB] OR gerbillinae[TIAB] OR gerbil[TIAB] OR gerbils[TIAB] OR jird[TIAB] OR jirds[TIAB] OR merione[TIAB] OR meriones[TIAB] OR rabbits[TIAB] OR rabbit[TIAB] OR hares[TIAB] OR hare[TIAB] OR diptera[TIAB] OR flies[TIAB] OR fly[TIAB] OR dipteral[TIAB] OR drosphila[TIAB] OR drosophilidae[TIAB] OR cats[TIAB] OR cat[TIAB] OR carus[TIAB] OR felis[TIAB] OR nematoda[TIAB] OR nematode[TIAB] OR nematoda[TIAB] OR nematode[TIAB] OR nematodes[TIAB] OR sipunculida[TIAB] OR dogs[TIAB] OR dog[TIAB] OR canine[TIAB] OR canines[TIAB] OR canis[TIAB] OR sheep[TIAB] OR sheeps[TIAB] OR mouflon[TIAB] OR mouflons[TIAB] OR ovis[TIAB] OR goats[TIAB] OR goat[TIAB] OR capra[TIAB] OR capras[TIAB] OR rupicapra[TIAB] OR chamois[TIAB] OR haplorhini[TIAB] OR monkey[TIAB] OR monkeys[TIAB] OR anthropoidea[TIAB] OR anthropoids[TIAB] OR saguinus[TIAB] OR tamarin[TIAB] OR tamarins[TIAB] OR leontopithecus[TIAB] OR hominidae[TIAB] OR ape[TIAB] OR apes[TIAB] OR pan[TIAB] OR paniscus[TIAB] OR "pan paniscus"[TIAB] OR bonobo[TIAB] OR bonobos[TIAB] OR troglodytes[TIAB] OR "pan troglodytes"[TIAB] OR gibbon[TIAB] OR gibbons[TIAB] OR siamang[TIAB] OR siamangs[TIAB] OR nomascus[TIAB] OR symphalangus[TIAB] OR chimpanzee[TIAB] OR chimpanzees[TIAB] OR prosimians[TIAB] OR "bush baby"[TIAB] OR prosimian[TIAB]			

	OR bush babies[TIAB] OR galagos[TIAB] OR galago[TIAB] OR pongidae[TIAB] OR gorilla[TIAB] OR gorillas[TIAB] OR pongo[TIAB] OR pygmaeus[TIAB] OR "pongo pygmaeus"[TIAB] OR orangutans[TIAB] OR pygmaeus[TIAB] OR lemur[TIAB] OR lemurs[TIAB] OR lemuridae[TIAB] OR horse[TIAB] OR horses[TIAB] OR pongo[TIAB] OR equus[TIAB] OR cow[TIAB] OR calf[TIAB] OR bull[TIAB] OR chicken[TIAB] OR chickens[TIAB] OR gallus[TIAB] OR quail[TIAB] OR bird[TIAB] OR birds[TIAB] OR quails[TIAB] OR poultry[TIAB] OR poultries[TIAB] OR fowl[TIAB] OR fowls[TIAB] OR reptile[TIAB] OR reptilia[TIAB] OR reptiles[TIAB] OR snakes[TIAB] OR snake[TIAB] OR lizard[TIAB] OR lizards[TIAB] OR alligator[TIAB] OR alligators[TIAB] OR crocodile[TIAB] OR crocodiles[TIAB] OR turtle[TIAB] OR turtles[TIAB] OR amphibian[TIAB] OR amphibians[TIAB] OR amphibia[TIAB] OR frog[TIAB] OR frogs[TIAB] OR bombina[TIAB] OR salientia[TIAB] OR toad[TIAB] OR toads[TIAB] OR "epidalea calamita"[TIAB] OR salamander[TIAB] OR salamanders[TIAB] OR eel[TIAB] OR eels[TIAB] OR fish[TIAB] OR fishes[TIAB] OR pisces[TIAB] OR catfish[TIAB] OR catfishes[TIAB] OR siluriformes[TIAB] OR arius[TIAB] OR heteropneustes[TIAB] OR sheatfish[TIAB] OR perch[TIAB] OR perches[TIAB] OR percidae[TIAB] OR perca[TIAB] OR trout[TIAB] OR trouts[TIAB] OR char[TIAB] OR chars[TIAB] OR salvelinus[TIAB] OR "fathead minnow"[TIAB] OR minnow[TIAB] OR cyprinidae[TIAB] OR carps[TIAB] OR carp[TIAB] OR zebrafish[TIAB] OR zebrafishes[TIAB] OR goldfish[TIAB] OR goldfishes[TIAB] OR guppy[TIAB] OR guppies[TIAB] OR chub[TIAB] OR chubs[TIAB] OR tinca[TIAB] OR barbels[TIAB] OR barbus[TIAB] OR pimephales[TIAB] OR promelas[TIAB] OR "poecilia reticulata"[TIAB] OR mullet[TIAB]			

	OR mullets[TIAB] OR seahorse[TIAB] OR seahorses[TIAB] OR mugil curema[TIAB] OR atlantic cod[TIAB] OR shark[TIAB] OR sharks[TIAB] OR catshark[TIAB] OR anguilla[TIAB] OR salmonid[TIAB] OR salmonids[TIAB] OR whitefish[TIAB] OR whitefishes[TIAB] OR salmon[TIAB] OR salmons[TIAB] OR sole[TIAB] OR solea[TIAB] OR "sea lamprey"[TIAB] OR lamprey[TIAB] OR lampreys[TIAB] OR pumpkinseed[TIAB] OR sunfish[TIAB] OR sunfishes[TIAB] OR tilapia[TIAB] OR tilapias[TIAB] OR turbot[TIAB] OR turbots[TIAB] OR flatfish[TIAB] OR flatfishes[TIAB] OR sciuridae[TIAB] OR squirrel[TIAB] OR squirrels[TIAB] OR chipmunk[TIAB] OR chipmunks[TIAB] OR suslik[TIAB] OR susliks[TIAB] OR vole[TIAB] OR voles[TIAB] OR lemming[TIAB] OR lemmings[TIAB] OR muskrat[TIAB] OR muskrats[TIAB] OR lemmus[TIAB] OR otter[TIAB] OR otters[TIAB] OR marten[TIAB] OR martens[TIAB] OR martes[TIAB] OR weasel[TIAB] OR badger[TIAB] OR badgers[TIAB] OR ermine[TIAB] OR mink[TIAB] OR minks[TIAB] OR sable[TIAB] OR sables[TIAB] OR gulo[TIAB] OR gulos[TIAB] OR wolverine[TIAB] OR wolverines[TIAB] OR minks[TIAB] OR mustela[TIAB] OR llama[TIAB] OR llamas[TIAB] OR alpaca[TIAB] OR alpacas[TIAB] OR camelid[TIAB] OR camelids[TIAB] OR guanaco[TIAB] OR guanacos[TIAB] OR chiroptera[TIAB] OR chiropteras[TIAB] OR bat[TIAB] OR bats[TIAB] OR fox[TIAB] OR foxes[TIAB] OR iguana[TIAB] OR iguanas[TIAB] OR xenopus laevis[TIAB] OR parakeet[TIAB] OR parakeets[TIAB] OR parrot[TIAB] OR parrots[TIAB] OR donkey[TIAB] OR donkeys[TIAB] OR mule[TIAB] OR mules[TIAB] OR zebra[TIAB] OR zebras[TIAB] OR shrew[TIAB] OR shrews[TIAB] OR bison[TIAB] OR bisons[TIAB] OR buffalo[TIAB] OR			

	buffaloes[TIAB] OR deer[TIAB] OR deers[TIAB] OR bear[TIAB] OR bears[TIAB] OR panda[TIAB] OR pandas[TIAB] OR "wild hog"[TIAB] OR "wild boar"[TIAB] OR fitchew[TIAB] OR fitch[TIAB] OR beaver[TIAB] OR beavers[TIAB] OR jerboa[TIAB] OR jerboas[TIAB] OR capybara[TIAB] OR capybaras[TIAB]) NOT medline[subset])			
	#1 AND #2 AND #3 AND #4	**439**	**15:09:15**	**04/10/2021**

Table S2

Supporting Information

S2 Table. General characteristics of the experimental models used in all studies included in this systematic review

Reference	Country	Strain	Sex	Age	Weight	Diabetics
Miller et al., 2017	USA	C57BL/6	F	10-12 wk	35-45 g	Genetic
Wong et al., 2019	MAS	SpragueDawley	M	?	200-250g	Induced
Seitz et al., 2010	GER	C57BL/6J	F	6-12 wk	?	Induced
Yan et al., 2018	CHN	ICR	M	6 wk	25g	Induced
Bazrafshan et al., 2014	IRA	SpragueDawley	M	Adult	260-280g	Induced
Cifuentes et al., 2020	SPA	Wistar	F	Adult	183-260g	Induced
Sidhu et al., 1999	IND	C57BL-Swiss albino	F/M	8-10 wk	250–300g	Ind/gen
Wang et al., 2019	USA	BKS	F	11-12 wk	?	Genetic
Kim et al., 2008	JPN	BKS	M	10 wk	?	Genetic
Heit et al., 2012	USA	C57BL/ksj	M	8-10 wk	?	Genetic
Lin et al., 2015	CHN	Wistar	M	?	?	Induced
Demyanenko et al., 2017	RUS	C57BL/ksj	M	9 wk	?	Genetic
Kao et al., 2011	CHN	C57BL/J	M	10-12 wk	?	Genetic
Yan et al., 2020	CHN	C57BL/J	M	6 wk	?	Induced
Huang et al., 2016	JPN	C57BL/ksj	M	10 wk	?	Genetic
Cheing et al., 2014	CHN	SpragueDawley	M	8-10 wk	280-320g	Induced
Liu et al., 2020	CHN	C57BL/6	M	8 wk	?	Induced
Lee et al., 2016	KOR	C57BL/6	M	8 wk	?	Induced

GER = Germany, CHN = China, d = day, SPA= Spain, F = female, g= gram, IND = India, IRA= Iran, KOR = South Korea, M = male, MAL = Malaysia, JPN=Japan, RUS = Russia, USA = United States of America,
wk = week.

Table S3

Supporting Information

Table S3. General characteristics of the wounds in all studies included in this systematic review

Wound							
**Animal model: murine model**							
**Reference**	**Assepsias**	**Biopsia day**	**Site**	**Size**	**Number of wounds for animal**	**Anesthesia (drug and dose)**	**Euthanasia (methods)**
Miller et al., 2017	?	0/3/7/14/21/28	D	8 mm	1	?	?
Wong et al., 2019	?	7/14	D	6 mm	4	?	?
Seitz et al., 2010	EtOH 70%	1/3/5/7/11	D	5 mm	6	Ket/Xyl ?	?
Yan et al., 2018	EtOH 70%	7/14	D	7 mm	1	Chloral hydrate (400 mg/kg)	Cervical deslocation
Bazrafshan et al., 2014	?	3/5/7	D	8 mm	4	?	?
Cifuentes et al., 2020	Sterilized dressing	3/7/14/21	D	15 mm	1	Ket (90 mg/kg)/ Xyl (10 mg/kg)	?
Sidhu et al., 1999	EtOH 70%	4/7/10	D	8 mm	6	Pentobarbitone (30 mg/kg)	?
Wang et al., 2019	?	1/4/7/10	D	8 mm	2	Isoflurane ?	?
Kim et al., 2008	EtOH 70%	7/14	D	10 mm^2	?	Pentobarbitone (30 mg/kg)	Pentobarbitone (30 mg/kg)
Heit et al., 2012	EtOH 70%	2/ 4/ 7	D	10 mm^2	?	Pentobarbitone (60 mg/kg)	?
Lin et al., 2015	?	12	D	6 mm	4	?	?
Demyanenko et al., 2017	EtOH 70%	1/3/5/ 7	D	7 mm	1	Zoletil 50mg/Kg	?
Kao et al., 2011	?	3/7/10/14/21/28	D	10 mm^2	?	?	?
Yan et al., 2020	?	1/5/8/14	D	9 mm	?	Avertin (240mg/Kg)	?
Huang et al., 2016	?	4/7/10/14/28	D	10 mm	?	Pentobarbitone ?	?
Cheing et al., 2014	EtOH 70%	7/10/14/21	D	20 mm	1	Ket (100 mg/kg)/ Xyl (10 mg/kg)	?
Liu et al., 2020	?	5/ 9	D	5 mm	1	Chloral hydrate ?	?
Lee et al., 2016	EtOH 70%	1/4/7/10	D	4 mm	1	?	?

EtOH = ethanol, D= Dorsal, Iso = Isoflurane, Ket = Ketamine, ? = Uninformed, Xyl = xylazine, Zolaz = Zolazep.

Table S4. Biases analyses (ARRIVE) of the studies.

**Title**	Miller et al., 2017	Wong et al., 2019	Seitz et al., 2010	Yan et al., 2018	Barzrafshan et al., 2014	Cifuentes et al., 2020	Sidhu et al., 1999	Wang et al., 2019	Kim et al., 2008	Heit et al., 2012	Lin et al., 2015	Demyanenko et al., 2017	Kao et al., 2011	Y. Yan et al., 2020	Huang et al., 2016	Cheing et al., 2014	Liu et al., 2020	Lee et al., 2016	
Accurate and concise description of the article content	✔	✔	✔	✔	✔	✔	✔	✔	✔	✔	✔	✔	✔	✔	✔	✔	✔	✔	
**Abstract**																			
Background summary, research objectives, methods, principal findings, and conclusions	✔							✔				✔	✔	✔	✔			✔	
**Introduction**																			
Sufficient scientific background	✔	✔	✔	✔	✔	✔	✔	✔	✔	✔	✔	✔	✔	✔	✔	✔	✔	✔	
Explanation of the experimental approach and rationale	✔	✔	✔	✔	✔	✔	✔	✔	✔	✔	✔	✔	✔	✔	✔	✔	✔	✔	
**Objectives**																			
Clear primary and second objectives	✔					✔							✔	✔				✔	
**Materials and Methods**																			
Nature of the ethical review permissions, relevant licenses and national or institutional guidelines for the care and use of animals	✔	✔	✔	✔	✔	✔	✔	✔	✔	✔	✔	✔	✔	✔	✔	✔	✔	✔	
Study design																			
Number of animals per group	✔	✔	✔	✔	✔	✔	✔	✔		✔		✔	✔	✔	✔	✔	✔		
Information on whether the experiment was performed as a blind controlled study																✔	✔		
Experimental procedures																			
Treatment Description	✔	✔		✔	✔	✔	✔	✔	✔	✔	✔	✔	✔	✔	✔	✔	✔	✔	
Treatment dosage	✔	✔		✔	✔	✔	✔	✔	✔	✔	✔	✔	✔	✔	✔	✔	✔	✔	
Treatment Duration		✔		✔	✔	✔	✔	✔	✔	✔	✔	✔	✔			✔	✔	✔	

Time of day of treatment administration																			
Experimental animals																			
Information regarding animal species	✔	✔	✔	✔	✔	✔	✔	✔	✔	✔	✔	✔	✔	✔	✔	✔	✔	✔	
Animals’ strain																			
Animals’ sex	✔	✔	✔	✔	✔	✔	✔	✔	✔	✔	✔	✔		✔	✔	✔	✔	✔	
Animals’ Weight range	✔	✔	✔	✔	✔	✔	✔									✔			
Animals’ Age	✔		✔		✔		✔	✔	✔	✔		✔	✔	✔	✔	✔	✔	✔	
Description of genetic modification status (Knock-out, transgenic, SPF)	✔	✔	✔		✔	✔	✔	✔				✔		✔	✔	✔	✔	✔	
Information related to previous procedures performed on the animals																			
Housing and husbandry																			
Housing of experimental animals (facility type, cage or housing type, material, number of cage companions)									✔										
Husbandry conditions (breeding program, light/dark cycle, temperature, water)				✔	✔	✔										✔	✔		
Welfare-related assessments and interventions that were carried out before, during, or after the experiment																			
Sample size																			
Total number of animals used in each experimental and the number of animals in each experimental group	✔	✔		✔	✔	✔	✔	✔		✔	✔	✔	✔	✔	✔	✔	✔	✔	
Explanation regarding the decision of the number of animals and details of sample size calculation																			
Allocating animals to experimental groups																			
Full details of how animals were allocated to experimental groups (including randomization or matching)																			
Order in which the animals in the different experimental groups were treated and assessed																			
Experimental outcomes																			
Clear experimental outcomes assessed	✔	✔	✔	✔	✔	✔	✔	✔	✔	✔	✔	✔	✔	✔	✔	✔	✔	✔	
Statistical methods																			
Statistical methods used for each analysis	✔	✔	✔	✔	✔	✔	✔		✔	✔	✔	✔	✔	✔	✔	✔	✔	✔	

Unit of analysis specifications for each dataset		✓	✓	✓	✓	✓	✓		✓	✓	✓	✓	✓	✓	✓	✓	✓	✓	
Methods used to assess whether the data met the assumptions of the statistical approach		✓	✓	✓	✓	✓			✓	✓	✓	✓	✓	✓	✓	✓	✓	✓	
**Results**																			
Baseline data																			
Description of animals health status, for each experimental group, before treatment	✓			✓				✓	✓										
Number analyzed																			
Number or animals in each group included in each analysis (absolute numbers)	✓	✓	✓	✓	✓	✓	✓	✓		✓		✓	✓	✓	✓	✓	✓	✓	
Animals or data not included in the analysis (and explanation for the exclusion)																			
Outcomes and estimation																			
Information (Mean= Standard Deviation)	✓	✓	✓	✓	✓	✓	✓		✓	✓	✓	✓	✓	✓	✓	✓	✓	✓	
Adverse events																			
Information regarding mortality of experimental animals (Mean=Standard Deviation)																			
Modifications to the experimental protocols made to reduce adverse events																			
**Discussion**																			
Interpretation /scientific implications																			
Interpretation of the results, taking into account the study objectives and hypotheses, current theory and relevant studies	✓	✓	✓	✓	✓	✓	✓	✓	✓	✓	✓	✓	✓	✓	✓	✓	✓	✓	
Comments on the study limitations (sources of bias, limitations of the animal model, imprecision associated with the results)																		✓	
Generalisability /translation																			
Comments on how the findings are likely to translate to other species or systems, including relevance to human biology				✓	✓	✓	✓	✓	✓	✓	✓	✓	✓			✓		✓	
Funding																			
List of funding sources and the role of the funder(s) in the studt			✓	✓	✓	✓					✓	✓		✓	✓	✓	✓	✓	
Results of total	**21**	**20**	**18**	**23**	**24**	**24**	**21**	**19**	**19**	**20**	**18**	**23**	**21**	**22**	**21**	**25**	**23**	**24**	

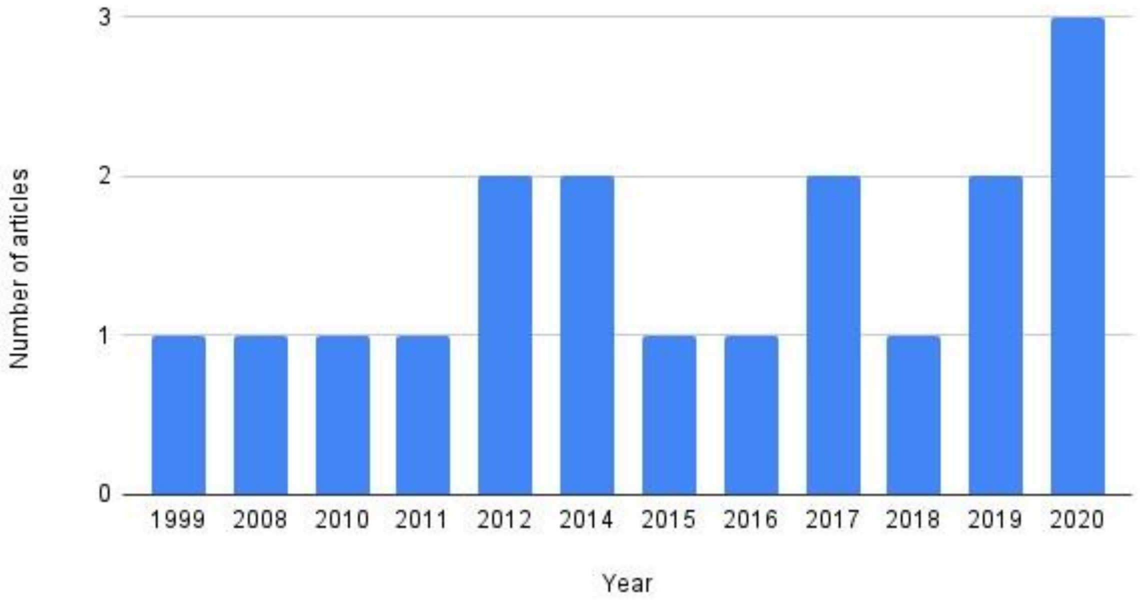

Figure S1: Number of publications per year.
